# A Meniscus Multifocusing Compound Eye Camera Based on Negative Pressure Forming Technology

**DOI:** 10.3390/mi14020420

**Published:** 2023-02-10

**Authors:** Xin Feng, Yongshun Liu, Junyu Dong, Yongjian Yu, Yi Xing, Fengfeng Shu, Lanxin Peng, Yihui Wu

**Affiliations:** 1 Changchun Institute of Optics, Fine Mechanics and Physics (CIOMP), Chinese Academy of Sciences, Changchun 130033, China; 2 University of Chinese Academy of Sciences, Beijing 100039, China; 3 School of Ophthalmology and Optometry and Eye Hospital, Wenzhou Medical University, Wenzhou 325035, China

**Keywords:** curved compound eye, ommatidium, multiple focal lengths, high resolution

## Abstract

To meet the challenge of preparing a high-resolution compound eye, this paper proposes a multi-focal-length meniscus compound eye based on MEMS negative pressure molding technology. The aperture is increased, a large field of view angle of 101.14° is obtained, and the ommatidia radius of each stage is gradually increased from 250 μm to 440 μm. A meniscus structure is used to improve the imaging quality of the marginal compound eye so that its resolution can reach 36.00 lp/mm. The prepared microlenses have a uniform shape and a smooth surface, and both panoramic image stitching and moving object tracking are achieved. This technology has great potential for application in many fields, including automatic driving, machine vision, and medical endoscopy.

## 1. Introduction

Compound eyes have the advantages of miniaturization, a large field of view, high integration, and high temporal resolution [1,2,3,4] that traditional optical systems cannot match. In recent years, researchers have developed a variety of compound eye processing technologies, such as photolithography, chemical etching, and two-photon polymerization [5,6,7,8,9,10,11,12,13,14,15,16]. However, the development period of compound eye imaging systems has been comparatively short, and some technical obstacles are yet to be resolved, including low imaging resolution, and a production process that is long and complicated. New methods are therefore needed to address these problems. With the development of MEMS (micro-electro-mechanical systems) technology, researchers have achieved notable results in studies of bionic compound eyes. The resolution levels of compound eyes prepared by MEMS [17,18,19,20,21,22,23,24,25,26,27,28,29,30,31,32,33,34,35,36] technology have greatly increased, and many more scenes can now be applied. Such technologies have greatly promoted compound eye research and development, and microlens-based technologies such as artificial compound eyes are now widely used in endoscopy, robotic vision, 3D imaging, and navigation [37,38,39,40,41,42,43,44,45].

As research on curved compound eyes has advanced, demand for improved resolution has also risen. Because its focal lengths are unified in the same plane, the curved compound eye can obtain multichannel images, and high-quality images with a large field of view can also be obtained. The multifocusing lens is well suited to solving the problem of direct matching between a curved compound eye and a flat image sensor so that the ommatidia at different positions of the compound eye can converge in the same plane. Such structures are not only small in size, but also can obtain a large field of view. The BAC eye prepared by Dai [46] et al. using microfluidic-manipulation 3D printing technology can track the position of objects in three-dimensional space. Shi [47] et al. added a relay optical path between the compound eye and the CMOS, to extend the field of view angle to 122.4°. The hexagonal integrated meniscus multifocusing compound eye lens fabricated by Liang [48] et al. using the jet printing method achieved a field angle of 92.6° with a thickness of only 3.04 mm. However, when a compound eye lens is prepared by jet printing technology and thermal reflow methods, due to the influence of liquid tension on the surface, the size of the ommatidia cannot be enlarged, resulting in low resolution. In the previous study from our research group, non-contact polymer hot embossing [49] was used to prepare a meniscus multifocusing compound eye. This method is simple and fast, but the contours of the ommatidia are formed by the surface tension transition of the polymer at the glass transition temperature. Because of this, the limit of surface tension cannot be satisfied by the height, and the maximum resolution of the compound eye obtained by this method is 8.95 lp/mm. The imaging effect of this compound eye system, therefore, needed to be further improved. In the study described here, we obtained such an improvement.

**Figure 1:**
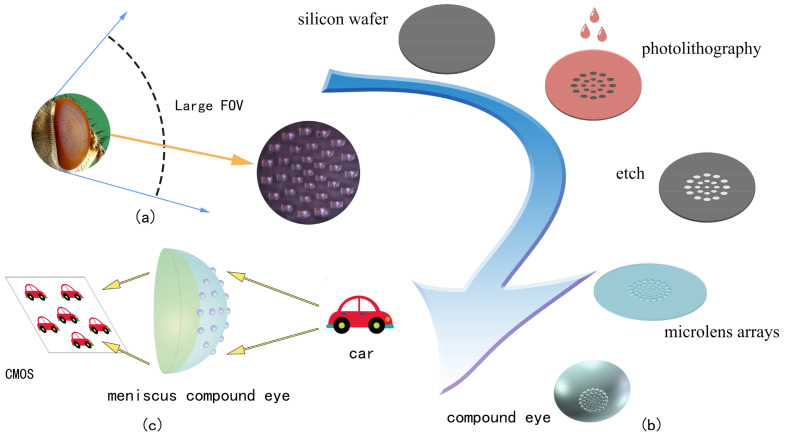
(**a**) Working principles of a compound eye. (**b**) Schematic illustration of the design principles of the meniscus compound eye. (**c**) The regulation principle of the meniscus compound eye.

In this paper, we present a fabrication method for a meniscus compound eye lens based on MEMS negative pressure forming technology, as shown in Figure 1. The compound eye lens is prepared by changing the pressure of the PDMS film covering the microporous array. The use of the meniscus structure reduces the reflection of light inside the substrate, improving the imaging effect. A total of 50 ommatidia are arranged according to the number of turns, the radius of the ommatidia is gradually increased from 250 μm to 440 μm, and the focal length is increased from 0.7 mm to 1 mm. The captured images can be stitched using Harris corner methods to achieve wide-angle panoramic images. Alternatively, a Gaussian mixture model (GMM) can be used to track moving objects. The method proposed in this paper allows for the speedy preparation of a compound eye lens, using a simplified preparation process, and may be further developed in future research for the purposes of three-dimensional imaging and target tracking.

## 2. Materials and Methods

### 2.1. Design of the Meniscus Multifocusing Compound Eye Lens

**Figure 2:**
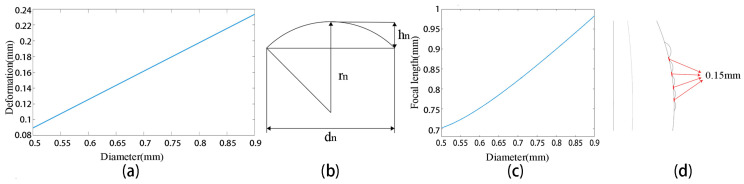
(**a**) The relationship between PDMS membrane deformation and diameter. (**b**) The relationships between the curvature radius *r_n_*, height *h_n_*, and diameter *d_n_* of the ommatidia (**c**) The relationship between ommatidia diameter and height. (**d**) The distance between adjacent ommatidia is 0.15 mm.

The performance of a compound eye system is determined by its design. In this study, we first designed the base of the compound eye with a radius of 9 mm and a height of 4.2 mm, enabling the covering of the target surface of a 1-inch flat image sensor. Under the same air pressure conditions, the tensile deformation of the polydimethylsiloxane (PDMS, Dow Chemical, China) membrane [50,51,52] and the diameter are approximately linear. In order to express the amount of deformation more intuitively, it can be drawn as a line graph, as in Figure 2a. Based on this principle, the negative pressure forming method can be used to design each ring of ommatidia of the meniscus compound eye.

In order for the compound eye to match the flat image sensor directly, the focal lengths of the ommatidia need to converge on the same plane. The focal lengths and the radii of the ommatidia increase with distance from the center. The main optical axis of all microlenses passes through the center of the curved base. The focal length *f_n_* of each ommatidium should be equal to the distance *ln* from the ommatidia to the focal plane, and this itself is mainly determined by the refractive index of the material *n* and the radius of curvature *r_n_*, as follows:

(1) $$f_{n}=l_{n}=\frac{r_{n}}{n-1}$$

In this study, the material is solidified with NOA63 (NORLAND, Jamesburg, NJ, USA), which has a refractive index n of 1.56. The relationship between the ommatidia curvature radius *r_n_*, height *h_n_*, and diameter *d_n_* can now be expressed algebraically (Equation (2)) and plotted visually (Figure 2b).

(2) $$r_{n}=\frac{d_{n}^{2}+4h_{n}^{2}}{8h_{n}}$$

By considering the PDMS membrane deformation, combined with *f_n_* and *r_n_*, the relationship between the radius of the ommatidia and the focal length of the ommatidia can now be obtained, as shown in Figure 2c.

In order to avoid the overlap of images from different ommatidia and taking into account the insufficient strength of the silicon wafer covered by the PDMS membrane during negative pressure forming, we set a distance of 0.15 mm between adjacent ommatidia, as shown in Figure 2d. The diameters, heights, and focal lengths of the ommatidia are all given in Table 1.

The larger the numerical aperture, the greater the amount of luminous flux entering the lens; this is proportional to the effective diameter of the lens and inversely proportional to the distance of the focal point. We can therefore calculate the numerical aperture of the central ommatidia, NA = 0.52, using the following formula:

(3) $$\mathrm{NA}=n\times \frac{r}{\sqrt{r^{2}+f^{2}}}$$

**Figure 3:**
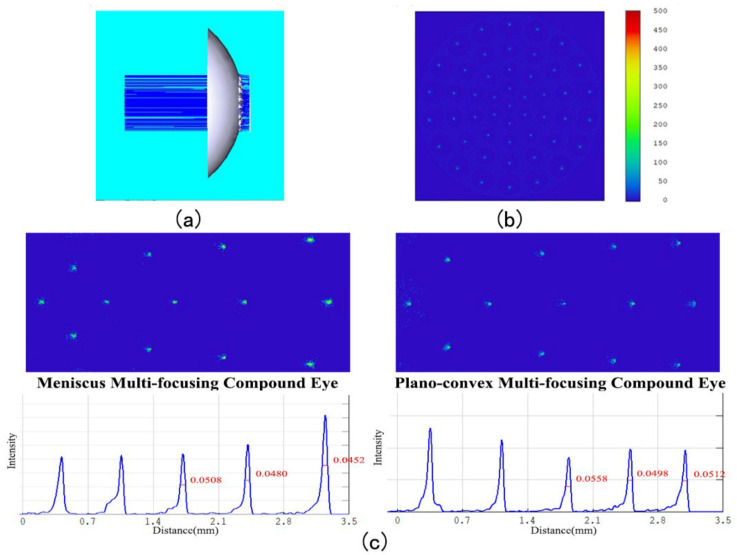
(**a**) Compound eye ray tracing. (**b**) The intensity distribution of the meniscus multifocusing compound eye. (**c**) Focal diagram and FWHM of meniscus compound eye and plano-convex compound eye at an incident angle of 50 degrees.

Next, we establish a corresponding compound eye model using SolidWorks software, with imported Zemax optics software for ray tracing purposes. Both spot size and light intensity are uniform. As can be seen in Figure 3a,b, the eye can be directly connected to the flat image sensor without the need for an optical relay, and FWHM (full width at half maximum) can be used as an evaluation index for imaging resolution. When the incident angle is 50 degrees, the distortions and FWHMs of edge ommatidia with the meniscus structure are better than those with the plano-convex structure, as shown in Figure 3c. The two most central circles of ommatidia cannot image a field of view beyond 50 degrees, so FWHMs are given only for the three circles of ommatidia furthest from the center.

### 2.2. Preparation of Multifocal Meniscus Compound Eye

**Figure 4:**
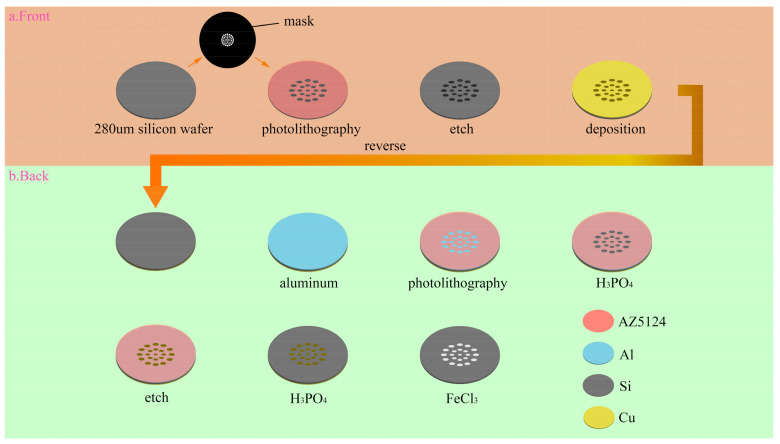
Micropore array silicon wafer preparation process.

First, we choose a silicon wafer with a thickness of 280 μm for photolithography purposes. Because holes are to be formed in the silicon, the wafer should be as thin as possible; however, too thin a silicon wafer will cause mechanical fracture during the negative pressure forming process. The positive photoresist AZ5214 (Merck, Weiterstadt, Germany) is used to obtain the pattern on the mask. The front surface of the silicon wafer is etched to a depth of 80 μm using the ICP (Alcatel 601E, Tours, France) plasma etching machine, and micropores are obtained by etching 200 μm on the back. Before etching the back, it is necessary to protect the etched front side. To this end, a copper film of 1 μm thickness is plated on the front side using a magnetron sputtering machine (Lesker LabLine, Jefferson Hills, PA, USA). The copper is soft and easy to remove. During the copper deposition process, the vacuum degree must be kept below 10^−8^ torr, otherwise, the copper film will start to detach. After the copper deposition is complete, it needs to stand for 24 h to release the stress. Because the etching depth on the back surface reaches 200 μm, the photoresist film does not prevent the back surface from being etched, and aluminum has better anti-etching qualities. For this reason, a layer of aluminum mask protection structure is plated onto the back surface. Photolithography is then performed on this surface. After the development is completed, a small amount of H_3_PO_4_ is used to remove the aluminum on the back structure, so that a back pattern with an aluminum mask is obtained. The back is then etched to a depth of 200 μm, and a silicon wafer structure with holes is obtained. FeCl_3_ is then used to remove the copper film, and H_3_PO_4_ is used to remove the remaining aluminum film on the back. By such means, a silicon wafer structure with a micropore array is obtained, and Figure 4 offers a visual presentation of the entire preparation process.

**Figure 5:**
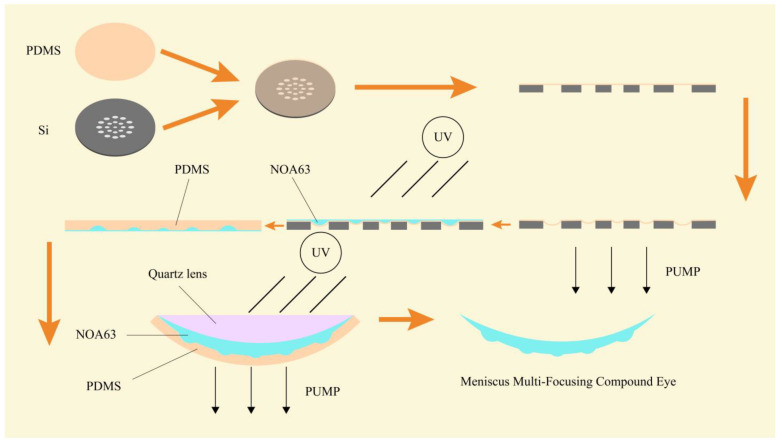
Meniscus multifocusing compound eye lens preparation process.

The PDMS membrane on the silicon wafer is now covered. Next, a negative pressure operation is performed on the lower side of the silicon wafer. Under the action of negative pressure, the PDMS membrane deforms, leaving the negative pressure on the lower side unchanged. NOA63 is then dripped onto the PDMS membrane and allowed to cure under ultraviolet light for 3 min. After waiting for cooling, a microlens array is obtained, which must be transformed into a curved surface. To achieve this, the PDMS is dropped onto the flat microlens and left in a horizontal position at 80 °C for 3 h. The PDMS is cured, and a PDMS mold is thereby obtained with a thickness of 3 mm and a structure of the opposite design to that of the flat microlens. The PDMS mold is then placed into the self-made mold with the pattern part facing upwards. A negative pressure operation is then performed. Under air pressure, the PDMS mold is deformed into a curved surface. The amount of surface deformation determines the base height of the meniscus multifocusing compound eye lens. Air pressure is maintained at a constant level, and NOA63 is allowed to drip. The quartz lens is then covered and cured for 7 min to obtain the final meniscus multifocusing compound eye lens, as shown in Figure 5.

### 2.3. Measurement and Characterization of Meniscus Multifocusing Compound Eye Lens

**Figure 6:**
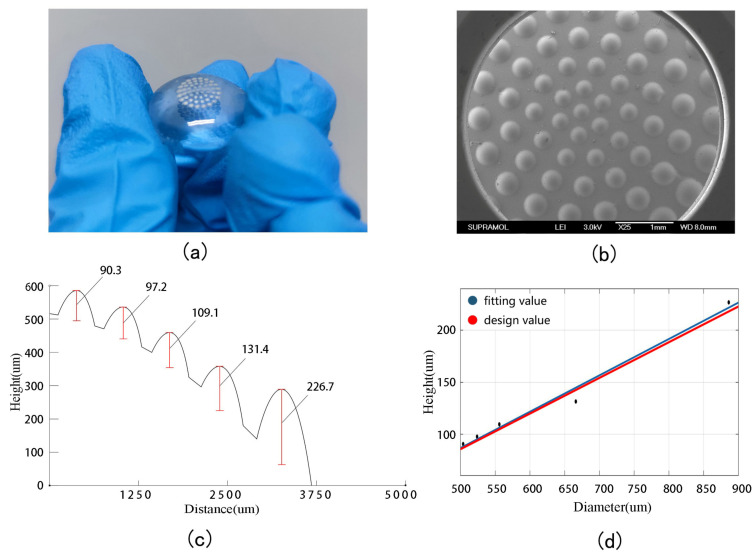
(**a**) A photo of the meniscus multifocusing compound eye lens. (**b**) A photo taken with a scanning electron microscope. (**c**) Profiler measurements of the height of the compound eye. (**d**) Compound eye height fit.

After the production of the compound eye lens, its geometric shape and optical performance can both be measured and characterized. Figure 6a is a photo of the compound eye lens taken by a camera. It can be seen that the surface of the lens is smooth and the light transmittance is good. Using a scanning electron microscope to take a picture of the lens as a whole, as shown in Figure 6b (JEOL FESEM 6700F electron microscope, Tokyo, Japan), the shape of a spherical coronal compound eye is clearly revealed.

A digital microscope (Keyence, Osaka, Japan) can now be used to precisely measure the diameter of each ring of the compound eye. In this study, beginning with the center ring and moving outwards, diameters are 504.06 μm, 523.99 μm, 555.99 μm, 666.11 μm, and 886.31 μm, respectively, were measured. These measurements were within 1% of the design theoretical values, and even this low level of discrepancy can be explained by the difference in the measurement accuracy of the instrument and the deformation of the PDMS membrane. Such small errors have no effect on the imaging quality, and so can be considered as consistent with the design.

Next, the height of each ring of the compound eye is measured with a profilometer. The pointer of the profilometer is drawn from the center of each ring, so that the height curve of the compound eye can be measured, as shown in Figure 6c. Beginning at the center ring and moving outwards, the heights of each ring were recorded as 90.3 μm, 97.2 μm, 109.1 μm, 131.4 μm, and 226.7 μm, respectively. Again, these measurements were within 1% of design theoretical values, and this low level of discrepancy can be explained by the difference in the deformation of the PDMS membrane and the inability of the pointer to accurately pass through the center of the compound eye. By fitting the height to the curve shown in Figure 6d, a linear relationship similar to that of design values can be obtained, in line with study expectations.

**Figure 7:**
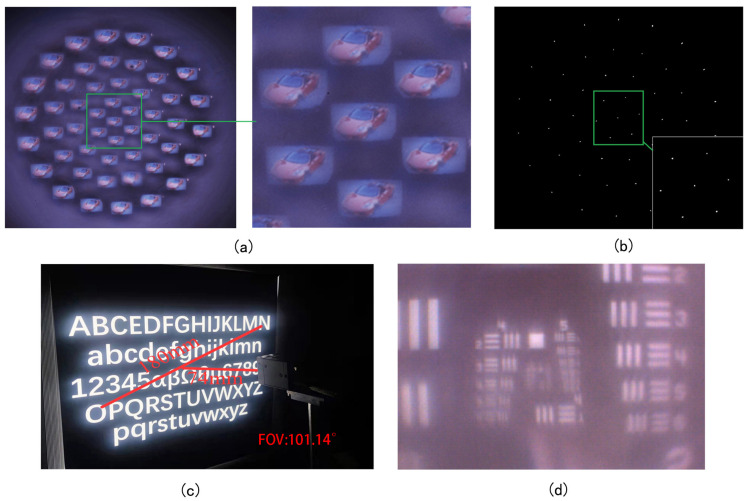
(**a**) Shoot results. (**b**) The focal spot of each ring. (**c**) Field angle measurement of compound eye camera. (**d**) Compound eye resolution test.

The meniscus multifocusing compound eye lens and CMOS (optical size: 1 inch, cell size: 2.4 × 2.4 μm, resolution: 5488 × 3672, frame rate: 19.5FPS) can now be assembled to form a camera without the optical relay, and its imaging effect tested using the photo of a car, as shown in Figure 7a. The laser (Edmund, wavelength: 632.8 nm) passes through the compound eye lens and, after testing and adjustment, the focal spot of each ring is obtained, confirming that the focal length is in the same plane, as shown in Figure 7b.

Next, a purpose-built device is used to measure the FOV (field of view) of the camera. As shown in Figure 7c, the camera is set symmetrically at the center of the image. The FOV of the compound eye lens is obtained using geometric relations and trigonometric functions. In a single shot, the distance between the furthest two letters is 180 mm, which is the value of *D*. *L* is the distance from the camera to the test chart letter. By calculation, the *FOV* of the multifocal meniscus compound lens is found to be 101.14°. This FOV can then be enlarged by increasing the number of circles of the compound eye.

(4) $$FOV=2arctan\frac{D}{2L}$$

In this study, the 1951 USAF resolution test chart (Thorlabs, Jessup, MD, USA) was used to test the resolution of the compound eye lens. The compound eye camera directly took pictures of the resolution chart, and the finest line width that the compound eye found was the second pair of the fifth group, as shown in Figure 7d. By consulting the parameters of the resolution board, the resolution of the ommatidia was then determined as 36.00 lp/mm. This high level of resolution is useful for subsequent image stitching and moving object recognition. High resolution also enables more accurate recognition of feature points, and of boundary selection between foreground and background.

### 2.4. System Application of Meniscus Multifocusing Compound Eye Lens

The imaging field angles of individual ommatidia are small. To obtain a large picture, it is necessary to stitch the obtained pictures of each channel. The overlapping images of the compound eye lens can meet the requirements of image stitching. Using the Harris corner detection method to stitch multiple images, the pixels in the area near the corners exhibit large changes in gradient direction and amplitude. When the pixel moves, the gray scale changes as follows:

(5) $$E\left(u,v\right)=\underset{x,y}{\sum }w\left(x,y\right){\left[I\left(x+u,y+v\right)-I\left(x,y\right)\right]}^{2}$$

where w (x, y) is the weight of the Gaussian function, and l (x, y) is the gradient of the gray value.

**Figure 8:**
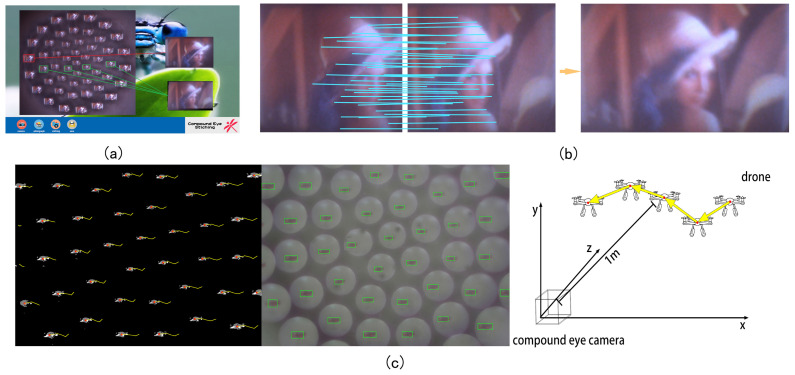
(**a**) Stitching software. (**b**) Schematic diagram of splicing. (**c**) Moving-drone tracking.

The corner points in the image can now be obtained. These can be selected by the ANMS (adaptive non-maximal suppression) method, and a specific number of key points are obtained. These can then be matched, and a new image obtained, as shown in Figure 8a,b.

One advantage of the compound eye is that it is sensitive to moving objects. The ability to track moving objects is therefore crucial. When the background is static, the moving object is in the foreground. The compound eye camera itself is in a static state, and the foreground and background are separated in real time on the captured image, to achieve the purpose of detecting moving objects. The GMM (Gaussian mixture model) has been extensively used because of its robustness in handling complex scenes and is able to meet the real-time requirements of the compound eye camera. Prior to shooting, a Gaussian mixture model was established for each pixel of the captured video. The number of Gaussian distributions is adaptive, and the background can be obtained by such means. In a long-term observation scene, the background occupies most of the time, and most of the data relate to background distribution. The GMM model is constantly updating and learning and can accommodate small disturbances. We set the background to black and the foreground to white to form a binary image separated from the foreground and the background, and perform contour recognition on the foreground image. We then use the compound eye camera to photograph a drone in flight. The distance from the drone to the camera is 1 m. The contour is drawn with a rectangle, the green frame is the recognized moving object, and the yellow curve is the trajectory of the recognized object. The picture shows the moving drone for five frames of images. The direction of movement and acceleration of the moving object can be judged according to the direction and length of the yellow line in the adjacent frame. The results shown in Figure 8c were obtained by averaging the drone positions identified by each channel and removing the results of excessively large and small values during the identification process. The large field of view and multi-channel imaging of the compound eye camera can accurately identify objects and implement corresponding functions in the event of partial image loss or low resolution.

**Figure 9:**
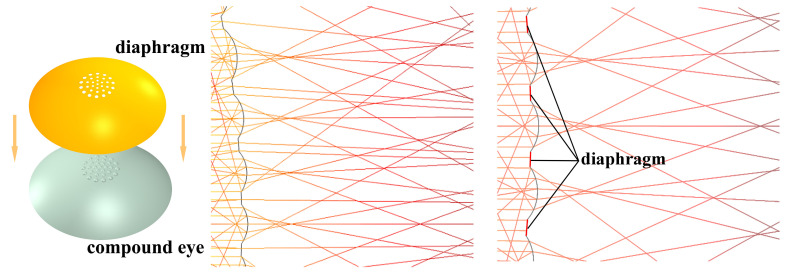
Comparison of light entering a compound eye lens with a diaphragm and a compound eye lens without a diaphragm.

The stray light in the spaces between the ommatidia reduces the imaging quality and the recognition rate of image stitching feature points and, consequently, GMM moving object recognition. In this study, we investigated the addition of diaphragms between adjacent ommatidia. To this end, Comsol software was used to obtain the results depicted in Figure 9. The use of a diaphragm resulted in a higher signal-to-noise ratio for imaging purposes. This improvement can result in higher imaging quality, so that compound eyes may be used in more fields.

## 3. Conclusions

In this paper, a fast and simple method for fabricating a meniscus multifocusing compound eye lens using MEMS negative pressure molding technology is proposed. This method uses the negative pressure deformation of the film to precisely control the shape of the ommatidia and enables the generation of panoramic imaging. By preparing MLA and transferring it to a meniscus substrate, the latter is able to effectively reduce the reflection of light inside the substrate, so that the field of view of the compound eye lens is enlarged, and imaging quality is also improved. The resolution of the ommatidia can reach up to 36.00 lp/mm. The field of view can also be increased by increasing the number of ommatidia circles. The size and height of ommatidia are designed so that their focal lengths are in the same plane and can be directly connected to the plane image sensor, reducing both the relay optical path and the volume of the system. A total of 50 images of different viewing angles are formed on the CMOS, and these independent images can form a high-resolution panoramic image. In addition, using Harris corner methods and the GMM algorithm, image stitching and the tracking of moving objects are realized, which may help researchers to study and test the imaging mechanisms and motion-tracking abilities of natural compound eyes. In conclusion, the unique characteristics of the compound eye lens described here have wide potential applications in microrobotics, medical testing, industrial testing, and other fields.

## Figures and Tables

**Table 1:** Diameters, heights, and focal lengths of the ommatidia.

Unit (mm)	Diameter *d_n_*	Height *h_n_*	Focal Length *f_n_*
1	0.500	0.090	0.709
2	0.520	0.097	0.717
3	0.560	0.109	0.736
4	0.660	0.131	0.801
5	0.880	0.226	0.980
